# Efficient Second- and Third-Harmonic Generations in Er^3+^/Fe^2+^-Doped Lithium Niobate Single Crystal with Engineered Surficial Cylindrical Hole Arrays

**DOI:** 10.3390/nano13101639

**Published:** 2023-05-14

**Authors:** Caixia Xu, Hongli Wu, Yanwei He, Long Xu

**Affiliations:** 1 School of Primary Education, Chongqing Normal University, Chongqing 400700, China; 2 School of Physics and Electronic Engineering, Chongqing Normal University, Chongqing 401331, China; 3 Department of Electrical and Computer Engineering, University of California, Riverside, CA 92521, USA; 4 Chongqing Key Laboratory of Micro&Nano Structure Optoelectronics, School of Physical Science and Technology, Southwest University, Chongqing 400715, China

**Keywords:** lithium niobate, second-harmonic generation, third-harmonic generation, periodic cylindrical pit arrays, femtosecond laser ablation

## Abstract

Herein, significant enhancement of second- and third-harmonic generation efficiencies in a 1 mol% Er^3+^ and 0.07 mol% Fe^2+^-doped lithium niobate single-crystal plate were achieved after ablating periodic cylindrical pit arrays on the surface. Enhanced absorption and reduced transmittance of light were measured when the incident light signal passed through the patterned sample. Enhanced photoluminescence and two-photon-pumped upconversion emission spectra were also explored to obtain more details on the efficiency gains. The excitation-energy-dependent second-harmonic generation efficiency was measured, and an enhancement as high as 20-fold was calculated. The conversion efficiency of second-harmonic generation is 1 to 3 orders higher than that from other lithium niobite metasurfaces and nanoantennas. This work provides a convenient and effective method to improve the nonlinear conversion efficiency in a thin lithium niobite plate, which is desirable for applying to integrated optical devices.

## 1. Introduction

The optical nonlinearity of single-crystal materials is widely used in various fields, such as lasers at various wavelengths [1], optical frequency conversion [2], photorefraction [3,4], optical energy storage [5,6], all-optical switches [7,8,9,10], optical focusing and defocusing [11], and optical communications [12]. Second-harmonic generation (SHG) and third-harmonic generation (THG) have attracted a great deal of attention in the development of high-performance lasers and new light sources [13,14,15,16,17]. With the improvement in crystal growth technology, many single-crystal materials have become ideal candidates for nonlinear optical crystals. Frequency conversion nonlinear laser crystals have been widely used in a variety of commercial lasers with high conversion efficiency [18], and the SHG and THG generated by the microscale periodic arrays have bright application prospects in integrated photonics [19,20]. Due to phase mismatching, no length optimizations, and low conversion efficiency as a result, many technologies and methods are used to increase the possibility of producing SHG and THG, and gradually increase their conversion efficiency [21,22,23]. From the nano- and micro-perspective, highly efficient SHG and THG in GaAs, AlGaAs, and Si nanoantennas, nanoresonators, and metasurfaces have been widely studied. SHG and THG in individual AlGaAs nanoantennas using far-field mapping with radially and azimuthally polarized cylindrical vector beams were achieved by Camacho-Morales and coworkers [24]. SHG in a Si metasurface strained by a SiN_x_ overlayer was demonstrated by Zhao and coworkers [25], where the SiN_x_ overlayer was used to break the inversion symmetry of crystalline Si.

Due to the large nonlinear polarizability, wide optical window, excellent nonlinearity, and electro-optical and piezoelectric properties, LiNbO_3_ single-crystal and thin films are also widely used in laser medical treatment, optical communication, laser processing, and holography [26,27,28,29]. In terms of second- and third-harmonic generations, picosecond 531 nm green and 385 nm violet light generations were performed in a LiNbO_3_ optical superlattice by using the quasi-phase-matching technique [30], and efficient second- and third-order nonlinear processes were demonstrated by using a waveguide structure and ferroelectric-domain-inverted grating [31]. Efficient second-harmonic generation was investigated in low-loss LiNbO_3_ thin-film nanowaveguides machined using the direct dry etching technique with a normalized conversion efficiency of 41% W^−1^cm^−2^ [32]. Recently, outstanding works have been conducted on improving the second-harmonic efficiency by processing periodic micro–nano structures on the surface of lithium niobate [33,34,35]. Periodic metasurfaces and microstructures were fabricated using reactive ion etching (RIE), laser ablation, and photolithography methods, and Mie-type and Fano-type resonances enhanced second-harmonic generations effectively [36]. Self-induced second-harmonic and sum frequency emissions were generated by a continuous-wave laser at 980 nm and a nanosecond pulse laser at 1064 nm by processing their surface into periodic rectangular arrays under femtosecond laser ablation [37]. To date, excellent works related to the efficient second harmonic in microstructure-modified lithium niobate crystals have been reported; however, the third harmonic is not easy to observe because of its low efficiency and mismatching with second-harmonic generation. 

To improve the generation efficiency of the second and third harmonics, in this work, the second- and third-harmonic generation efficiencies in a 1 mol% Er^3+^ and 0.07 mol% Fe^2+^-doped lithium niobate single crystal were investigated. The surface of the single crystal was modified with periodic cylindrical pit arrays using the femtosecond laser ablation technique. Enhanced absorption, photoluminescence, and two-photon-pumped upconversion emission spectra were explored to compare the differences between ablated and nonablated single crystals. SHG could occur simultaneously from 1064 to 532 nm and 1550 to 775 nm. Moreover, THG was also measured from 1550 to 515 nm, which verified that the quasi-phase-matching condition was more tolerant. This work provides a simple and easy-to-fabricate method for realizing high-order frequency conversion devices in integrated optics.

## 2. Materials and Methods

**Figure 1:**
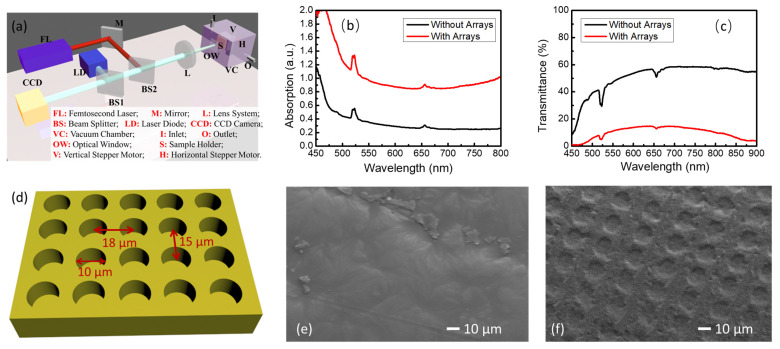
(**a**) Schematic of the optical path setup in femtosecond laser ablation system; (**b**) Room-temperature ground state absorption spectra of 1 mol% Er^3+^ and 0.07 mol% Fe^2+^-doped LiNbO_3_ single-crystal samples with and without pit array modification; (**c**) Transmittance spectra of 1 mol% Er^3+^ and 0.07 mol% Fe^2+^-doped LiNbO_3_ single-crystal samples with and without pit array modification; (**d**) Schematic diagram of the modified surface structure of the single-crystal plates; Surface morphology of the 1 mol% Er^3+^ and 0.07 mol% Fe^2+^-doped LiNbO_3_ single-crystal samples (**e**) without and (**f**) with periodic circular pit arrays taken with scanning electron microscope.

1 mol% Er^3+^ and 0.07 mol% Fe^2+^-doped LiNbO_3_ single-crystal samples were prepared by using the Czochralski method and were cut along the z-axis into a size of 10.0 mm × 10.0 mm × 0.1 mm. The surfaces of the single crystals were ablated into periodic cylindrical pit arrays using Yb^3+^: KGW femtosecond lasers with a center wavelength of 1030 nm, a frequency of 1 kHz, and a pulse width of 190 fs. As illustrated in Figure 1a, the high-power femtosecond laser beam was focused to a spot size of 20 μm with a focusing lens system, and the single-crystal samples were put on the focus point in a vacuum chamber. A white-light-emitting diode was collimated and overlapped with the femtosecond laser beam. The reflected light was collected and reached the CCD detector to observe the real-time processing of the sample surface. The positions of the samples were modulated with the horizontal and vertical stepper motors with a speed of 100 μm/s. High-purity nitrogen gas was pumped into the vacuum chamber as the protection gas. The energy of the pulse laser was set as 1.2 J/cm^2^, and the ablation area was 5.0 mm × 5.0 mm in the middle region of the plate. After the laser ablating, the machined samples were annealed at 800 K for 5 min in a nitrogen gas atmosphere in a rapid thermal annealing furnace. 

The morphology was captured using a scanning electron microscope (SEM, JEOL JSM-7100F, Tokyo, Japan). The ground state absorption spectrum of the samples was measured using a UV-Vis-IR absorption spectrometer (SHIMADZU Inc., Kyoto, Japan, UV-2600). A continuous-wave violet laser centered at 405 nm and near-infrared laser centered at 980 nm were used to irradiate the sample, and the photoluminescence and upconversion spectra were collected using a spectrometer (Ocean Optics Inc., QE65Pro, Shanghai, China). Second- and third-harmonic generations were investigated using a nanosecond laser centered at 1064 nm (500 µJ/5.0 kHz/20 ns) and a femtosecond laser centered at 1550 nm (33 nJ/93 fs/80 MHz, Calmar Laser Inc., Carmel X-1550, Palo Alto, Santa Clara, CA, USA), and the results were collected using the same spectrometer and an oscilloscope (Tektronix Inc., MD034, Beijing, China). 

## 3. Results and Discussion

The room-temperature ground state absorption and transmittance spectra of the samples with and without circular pit arrays were measured. The broadband halogen lamp was aligned using a collimating lens and the incident direction of the light beam was vertical with the surface of the Er^3+^/Fe^2+^-doped lithium niobate plate. As seen in Figure 1b,c, two typical absorption peaks were observed, corresponding to the stimulated absorption of photons as electrons were excited from the ground state to the excited state of ^2^H_11/2_ and ^4^F_9/2_ in the Er^3+^ ions. The absorption intensity of the sample with pit arrays increased in the broadband wavelength range, which is attributed to the resonant absorption of light within the periodic structures and the scattering of light by the irregular part of the surface [38,39]. The former dominated the increase in the absorption of light. Similarly, the transmission intensity of the patterned sample dropped significantly since most of the photons were absorbed and scattered away. Moreover, the resonance of the photons in the microstructure increased the effective thickness of the material. A schematic of the pit arrays is shown in Figure 1d. The cylindrical pits with a radius of 5.0 µm are arranged in an 18 × 15 µm grid. As seen in Figure 1e,f, the surface morphology of the 1 mol% Er^3+^ and 0.07 mol% Fe^2+^-doped LiNbO_3_ single-crystal samples with and without hole arrays was captured using a scanning electron microscope, and neat hole arrays could be seen on the surface of the modified sample.

**Figure 2:**
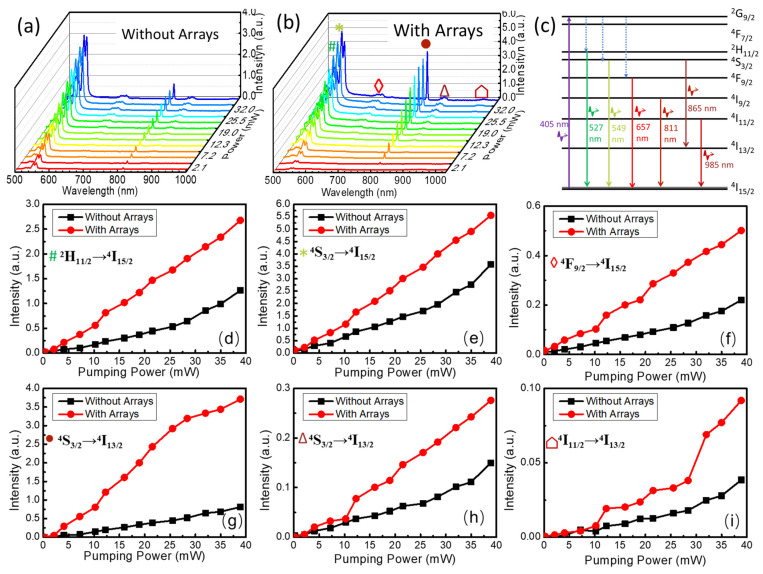
Photoluminescence emission spectra excited by the continuous-wave violet laser at 405 nm in the sample (**a**) with and (**b**) without circular hole array modification; (**c**) Schematic diagram of the energy level transition upon being irradiated by the violet laser. Changes in the luminescence intensity of photoluminescence emission peaks at (**d**) 527 nm, (**e**) 549 nm, (**f**) 657 nm, (**g**) 811 nm, (**h**) 865 nm, and (**i**) 985 nm along with the increase in the pumping power.

Figure 2a,b show the photoluminescence spectra stimulated using a 405 nm continuous-wave violet laser at different power. Upon the excitation of the pumping laser, six typical peaks were observed from both the original and patterned samples, which correspond to the energy transition of ^2^H_11/2_ to ^4^I_15/2_ (527 nm), ^4^S_3/2_ to ^4^I_15/2_ (549 nm), ^4^F_9/2_ to ^4^I_15/2_ (657 nm), ^4^I_9/2_ to ^4^I_15/2_ (811 nm), ^4^S_3/2_ to ^4^I_13/2_ (865 nm), and ^4^I_11/2_ to ^4^I_15/2_ (985 nm), respectively [37]. The energy level diagram is shown in Figure 2c. Figure 2d–i show the photoluminescence intensity of each peak from the patterned (red circular points) and original (black rectangular points) samples. As the power of the pumping laser increased, the photoluminescence intensity increased. By comparing the red and black lines, we can see that the photoluminescence is enhanced by an average of 200% after introducing the pit array, while the peak at 810 nm is enhanced by 6500%. It is worth noting that the emission peak at 810 nm has a very narrow full width at half maximum value (FWHM) of ~3 nm. By comparison, the FWHM of other emission peaks range between 10 and 25 nm. Two main factors played critical roles in the fluorescence enhancement: (1) the pit array enhanced the absorption efficiency of the laser light, especially at the wavelength of 405 nm; (2) multiple reflections, scattering, and resonance of light within the periodic arrays were formed, and the optical thickness of the single crystal was increased, thus causing the enhancement of fluorescence in the samples [40,41].

**Figure 3:**
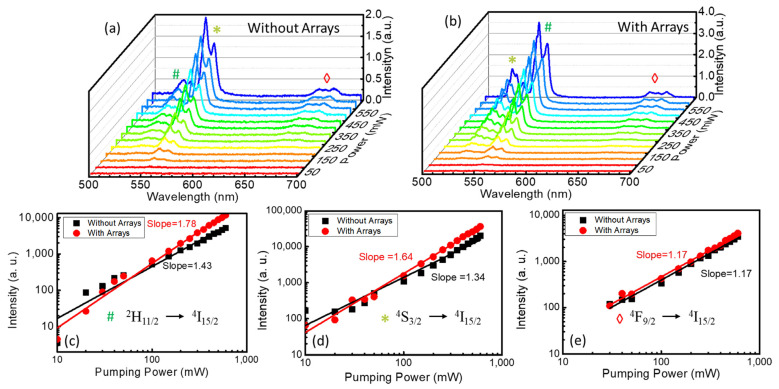
Two-photon-pumped upconversion emission spectra excited by the continuous-wave violet laser at 405 nm in the sample (**a**) with and (**b**) without circular hole array modification. Changes in the luminescence intensity of the two-photon-pumped upconversion emission peaks at (**c**) 527 nm, (**d**) 547 nm, (**e**) 657 nm along with the increase in the pumping power.

Figure 3a,b show the two-photon-pumped upconversion spectra stimulated by the continuous-wave near-infrared laser centered at 980 nm. In stark contrast to the fluorescence spectrum, the luminescence intensity of the upconversion spectrum was much weaker, and only three typical emission bands were observed, corresponding to the energy transition of ^2^H_11/2_ to ^4^I_15/2_ (527 nm), ^4^S_3/2_ to ^4^I_15/2_ (549 nm), and ^4^F_9/2_ to ^4^I_15/2_ (657 nm). In order to study the variation trend in upconversion luminescence in the single-crystal samples, the luminescence intensities of the two-photon-pumped upconversion emission peaks at 527 nm, 547 nm, and 657 nm were measured along with the increase in the pumping power. According to the law of multi-photon-pumped upconversion emission, the emitted light intensity is proportional to the nth power of the incident light intensity, that is, $I_{out}\propto I_{in}^{n}$, where *n* is the number of photons participating in the n-photon-pumped upconversion [42]. As shown in Figure 3c–e, *n* could be calculated based on the changing slopes of the emission peaks when both the *x* and *y* axes were plotted using logarithmic coordinates. It can be seen from the linear fitting of the experimental results that the slopes were all between 1 and 2. The slopes greater than 1 indicate that the detected spectra were the upconversion emission of two-photon pumping. The slopes less than 2 were due to the existence of many nonradiative transitions in the upconversion process. In addition, the luminescence slopes of the sample with microstructure arrays were larger than those without microstructures, because the gain brought by the microstructures compensated part of the nonradiative transition loss.

**Figure 4:**
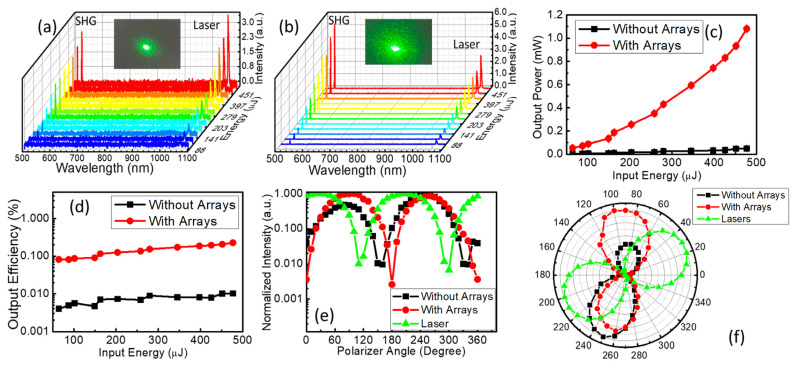
The spectra of fundamental frequency laser and SHG with the increase in excitation energy in (**a**) unpatterned sample and (**b**) surficial patterned sample (the intensity of the laser was attenuated by a bandpass filter at 532 nm, and the insets of (**a**,**b**) were the SHG beams recorded with a CCD camera); (**c**) The changes in output energy along with the increase in excitation energy in both patterned and unpatterned single crystals; (**d**) The SHG output efficiency variation under different excitation energy in both patterned and unpatterned single crystals; The linear polarization properties of the SHG and fundamental frequency laser in both patterned and unpatterned single crystals (**e**) in two-dimensional coordinates and (**f**) in polar coordinates.

From the analysis of photoluminescence and upconversion spectra, it is apparent that the microstructure arrays could be used to improve the luminescence performance of the Er^3+^- and Fe^2+^-ion-doped LiNbO_3_ single crystals significantly. To further study the enhancement of crystal performance by the periodic hole array structure, the second-harmonic generation (SHG) was studied using a nanosecond pulse laser centered at 1064 nm; the power and spectra of the SHG were recorded using a power meter and spectrometer at the same time (Refer to Figure S1 in Supplementary Information). As shown in Figure 4a,b and Figure S2, narrow second-harmonic generation spectra centered at 532.2 nm were observed with an FHWM of 1.4 nm, which was much narrower than that of the fundamental frequency laser (4.0 nm). SHG enhancements of more than 20-fold were found in samples with arrays of microstructures compared with those in unprocessed samples. The output power and efficiency of the SHG in the single crystals were measured; as seen in Figure 4c, the output power of the outgoing second harmonic increased with the pump laser energy. Then, the conversion efficiency of SHG could be calculated based on the efficiency conversion formula $\eta =\frac{P_{SHG}}{P_{FF}}$ [43], as shown in Figure 4d. The SHG conversion efficiency in the unpatterned single crystal increased with the increase in the fundamental frequency laser energy and reached 0.01% at 475 µJ, comparable to that of a GaAs metasurface [44]. With the increase in the fundamental frequency laser energy, the conversion efficiency of SHG in the patterned single crystal was maintained at a high efficiency of around 0.1–0.2% and increased slightly with excitation energy, which was 2 orders higher than that of the GaAs metasurface. In addition, compared with the SHG in lithium niobate metasurfaces, the conversion efficiency was 1 to 3 orders higher [23,36,45]. Combining the experimental results of photoluminescence and upconversion luminescence, the main factors contributing to this significant enhancement were discussed. Analogously, increased absorption and the effective thickness of the sample due to the periodic circular pit arrays contributed to the improved SHG emission. More importantly, the periodic structure made the propagation path of the photon more complicated, and the formation of the SHG more efficient with high tolerance through quasi-phase-matching (QPM) [46,47]. In the quasi-phase-matching process, the periodic hole arrays provided an additional photon momentum *k_QPM_*, which is proportional to the reciprocal of the periodic constant Λ. The existing *k_QPM_* improved the tolerance of the photon momentum conversion in the phase-matching process effectively [48]. Compared with the unetched single crystal, the second-harmonic laser speckle generated in the single crystal with the etched periodic microstructure was brighter and in a wider-spreading region, as shown in the inset of Figure 4a,b, which was the strong evidence for photonic resonance and complex transport pathways within the periodic arrays to enhance the QPM and SHG efficiency. In addition, the polarization characteristics of the green SHG emission were investigated, as seen in Figure 4e,f. An excellent linear polarization performance with the excitation ratio of 400:1 was obtained in the sample with periodic pit arrays, which was better than that of the fundamental frequency laser (149:1) and the SHG emission in the sample without a microstructure (104:1). The improved polarization of light was attributed to the resonance of photons in the periodic microstructure.

**Figure 5:**
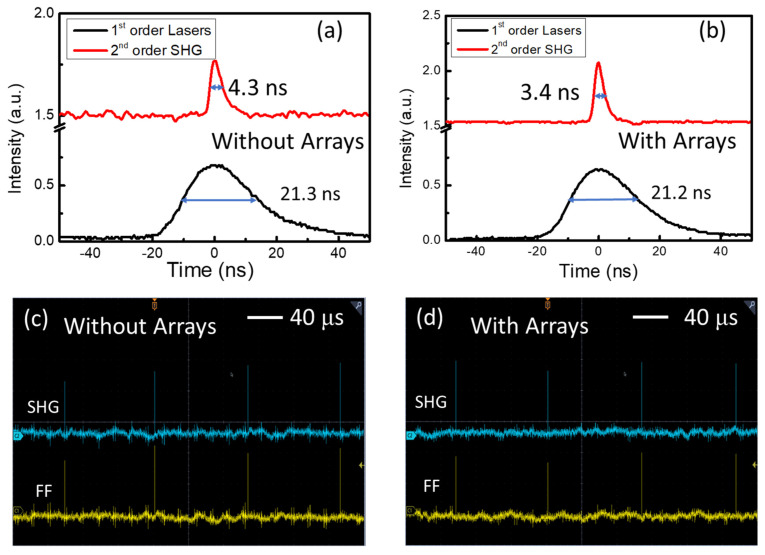
Dynamics of the fundamental frequency laser pulses and SHG pulses (**a**) in the unpatterned Er^3+^- and Fe^2+^-ion-doped LiNbO_3_ single-crystal plate and (**b**) in the surficial patterned sample; The dynamic changes in the fundamental frequency laser pulses (yellow) and the second-harmonic generations (green) with repeat laser pulses (**c**) in the unpatterned Er^3+^- and Fe^2+^-ion-doped LiNbO_3_ single-crystal plate and (**d**) in the surficial patterned sample.

The time domain dynamics of the SHG emission pulses at 532 nm were investigated using fast-response photodetectors (DET10A2, Rise time 1.0 ns, Thorlabs Inc., Shanghai, China) connected to an oscilloscope. As seen in Figure 5a,b, the full widths at half maximum (FWHMs) of the SHG emission pulses from the samples without and with a pit array were 4.3 ns and 3.4 ns, respectively, which are much narrower than that of the fundamental frequency laser (20 ns). Since the SHG conversion efficiency decreases monotonically with the laser energy, the pulse width of the SHG was narrower than the fundamental frequency laser when other factors were held constant. Dynamic stability and repeatability tests of the SHG at 532 nm were also carried out in both the samples, as seen in Figure 5c,d; stable and repeatable SHG can be observed from pulse to pulse, which is extremely important for its extension to optoelectronic and laser devices. 

**Figure 6:**
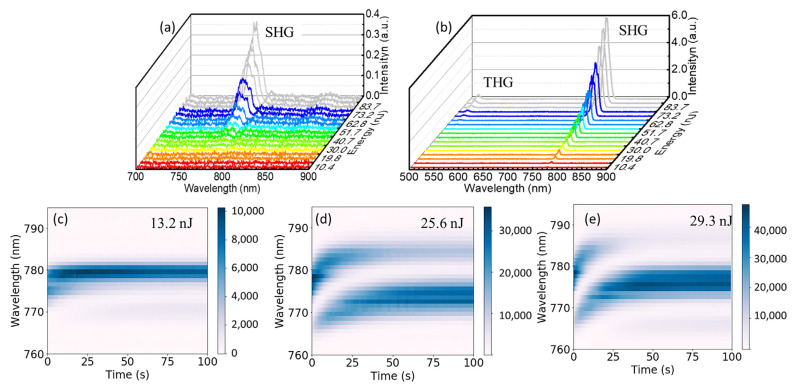
(**a**) The spectra of SHG with the increase in excitation energy in the unpatterned Er^3+^- and Fe^2+^-ion-doped LiNbO_3_ single-crystal plate; (**b**) The spectra of SHG and THG with the increase in excitation energy in the surficial patterned sample; The dynamics of the SHG modes upon the fundamental frequency femtosecond laser irradiated on the sample with etched arrays with an excitation energy of (**c**) 13.2 nJ, (**d**) 25.6 nJ, and (**e**) 29.3 nJ.

To further study the improvement in the optical nonlinear performance in the Er^3+^- and Fe^2+^-ion-doped LiNbO_3_ single crystal with a periodic pit array modification at other wavelengths, SHG and third-harmonic generations (THGs) were explored using a femtosecond laser at 1550 nm with a pulse width of 33 fs and pulse frequency of 80 MHz. As shown in Figure 6a, a broadband SHG emission centered at 775.0 nm with an FWHM of 15 nm was observed since the spectrum width of the fundamental frequency femtosecond laser at 1550 nm was wider than 30 nm. An enhancement as high as 16-fold in the SHG in the single-crystal sample with the etched periodic microstructures was measured, as seen in Figure 6b; the FWHM of the emission was 13 nm. Surprisingly, a THG with a central wavelength of 516.6 nanometers with an FWHM of 7.6 nm could be observed, although the conversion efficiency of the THG was much lower than that of SHG. The resonance and scattering of the ultrafast femtosecond laser pulses in the periodic microstructure effectively reduced the group velocity mismatching in the phase-matching process, thereby improving the conversion efficiency of SHG and harvesting THG. The THG of the 1064 nm light would appear at 355 nm but cannot be observed since its wavelength is in the absorption band of lithium niobate. In addition, multiple small peaks were observed spreading across the SHG spectrum, as seen in Figure 6b, especially when the excitation laser intensity was high. When the laser energy was weak (13.2 nJ), as shown in Figure 6c, only the SHG with a center wavelength of 780 nm exhibited a high conversion efficiency, and consequently, only one SHG mode was observed. When the laser energy was increased to 25.6 nJ, as seen in Figure 6d,e, more SHG modes appeared. The extremely high instantaneous power of the femtosecond laser made the broad-wavelength fundamental frequency laser meet the quasi-phase-matching conditions at many wavelengths, and these SHG modes competed and, finally, two SHG modes at 775 nm and 783 nm reached an equilibrium. As the laser energy was further increased to 29.3 nJ, the energy of the SHG mode at 775 nm increased rapidly and was much stronger than the other SHG peaks. The competition of these modes provided a deeper understanding of the formation of SHG modes in the periodic pit array microstructure and provided effective information for the future modulation of SHG laser modes.

**Figure 7:**
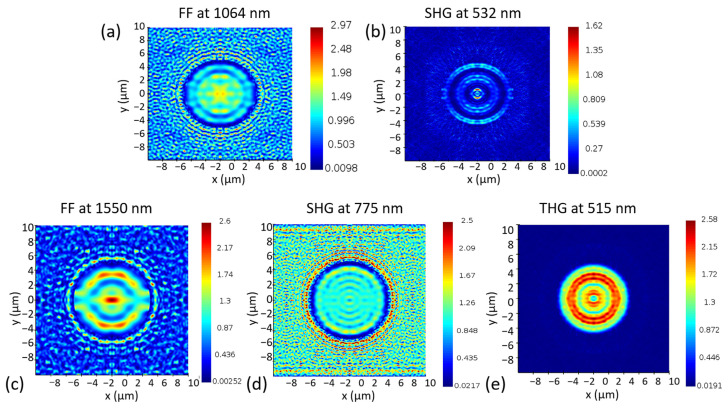
(**a**) Simulation result of the light intensity distribution of the fundamental frequency laser (FFL) at 1064 nm; (**b**) Simulation result of the light intensity distribution of the second-harmonic generation (SHG) at 532 nm; (**c**) Simulation result of the light intensity distribution of (**c**) the FFL at 1550 nm, (**d**) the SHG at 775 nm, and (**e**) the THG at 515 nm.

The light distribution of the fundamental frequency laser (FFL) at 1064 nm and the SHG at 532 nm were simulated using Lumerical FDTD solution software. The radius of the holes was set as 5.0 μm. The distances between the holes were set as 8.0 μm and 5.0 μm, respectively. Periodic boundaries were selected for the x-axis and y-axis, and the boundary of the z-axis was chosen as a perfectly matched layer (PML). The refractive index of the Er^3+^- and Fe^2+^-ion-doped LiNbO_3_ single crystals was built using the Sellmeier equation [49]. As seen in Figure 7a,b, the light intensity of the FFL was concentrated in the central holes and the gap between circular holes, while that of the second-harmonic generation was concentrated on the edge of the circular holes. The light distribution of the FFL at 1550 nm, SHG at 775 nm, and THG at 515 nm was also simulated, as seen in Figure 7c–e. Like the light distribution at 1064 nm, most of the FFL intensity at 1550 nm was distributed in the circular hole and between the cylindrical holes, and that of the SHG was primarily distributed in the space between the cylindrical holes, while the third-harmonic generation was mainly distributed within the cylindrical holes. The results of the simulations provide strong evidence for the generation of high-efficiency SHG and THG, which resulted from the resonance of light within and between cylindrical holes.

## 4. Conclusions

In conclusion, as enhancement in SHG as high as 20-fold was investigated in an Er^3+^- and Fe^2+^-doped lithium niobate single crystal with periodic circular hole arrays fabricated using the femtosecond laser ablation technique. The improved resonance and effective thickness resulted in an increment in absorption, photoluminescence, and two-photon-pumped upconversion emission efficiency. Efficient SHG centered at 532.2 nm with an FHWM of 1.4 nm was obtained, and the conversion efficiency was more than 0.2%, which was 2 orders higher than that of GaAs metasurfaces and 1 to 3 orders higher than that of other lithium niobate metasurfaces. An excellent linear polarization performance with an excitation ratio of 400:1 was achieved, originating from the resonance of photons in the periodic microstructure. Narrower SHG pulses with a FWHM of 3.4 ns were measured. In addition, multimode SHG at 775 nm and THG at 516 nm were observed simultaneously when excited by the femtosecond laser at 1550 nm. The multimode SHGs around 775 nm changed dynamically with the change in excitation energies. This work provides a deeper understanding of the formation and competition of SHG modes in the periodic pit array microstructure, which will be helpful in realizing high-order frequency conversion devices in integrated optics.

## Data Availability

Not applicable.

## Supplementary Materials

The following supporting information can be downloaded at: https://www.mdpi.com/article/10.3390/nano13101639/s1, Figure S1: The schematic of the optical path for SHG and THG measurement in Er^3+^/Fe^2+^ doped LiNbO_3_ thin films.; Figure S2: The spectra of fundamental frequency laser and SHG with the increase of excitation energy in (a) unpatterned sample and (b) surficial patterned sample.
