# Supplementary material for: Efficient Second- and Third-Harmonic Generations in Er3+/Fe2+-Doped Lithium Niobate Single Crystal with Engineered Surficial Cylindrical Hole Arrays
Source: Nanomaterials (Basel). 2023 May 14;13(10):1639. doi: 10.3390/nano13101639 (PMC10222391; doi:10.3390/nano13101639)
Supplement: Supplementary file 1 [file nanomaterials-13-01639-s001.zip › nanomaterials-2354122-supplementary.pdf]

Supplementary Information

Efficient Second- and Third-Harmonic Generations in Er^{3+}/Fe^{2+}-Doped Lithium Niobate Single Crystal with Engineered Surficial Cylindrical Hole Arrays

Caixia Xu [1,2], Hongli Wu [2], Yanwei He [3] and Long Xu [4,*]

1 School of Primary Education, Chongqing Normal University, Chongqing 400700, China
2 School of Physics and Electronic Engineering, Chongqing Normal University, Chongqing 401331, China
3 Department of Electrical and Computer Engineering, University of California, Riverside, CA 92521, USA
4 Chongqing Key Laboratory of Micro&Nano Structure Optoelectronics, School of Physical Science and Technology, Southwest University, Chongqing 400715, China
* Correspondence: longxu@swu.edu.cn

The second harmonic generation and third harmonic generation were measured by using the optical path in Fig. S1. The nanosecond/femtosecond laser was filtered by using a bandpass filter centered at 1064 nm/1550 nm. The first beam splitter (BS) was used to monitor the power/energy of the laser pulses. And the laser beam injected onto the surface of the sample vertically. Another BS was used to monitor the power of the SHG. Both the power/energy of the laser and SHG was recorded by using a power meter. And the spectra of SHG and THG were recorded using the spectrometer (QE65 Pro, Ocean Optics Inc.).

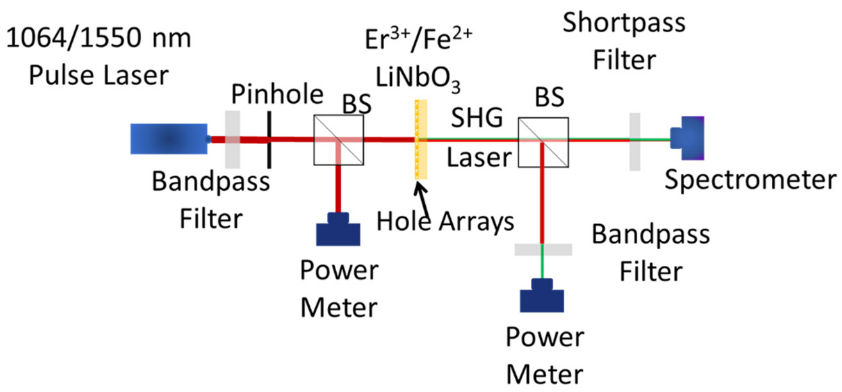

Figure S1 The schematic of the optical path for SHG and THG measurement in Er^{3+}/Fe^{2+} doped $LiNbO_3$ thin films.

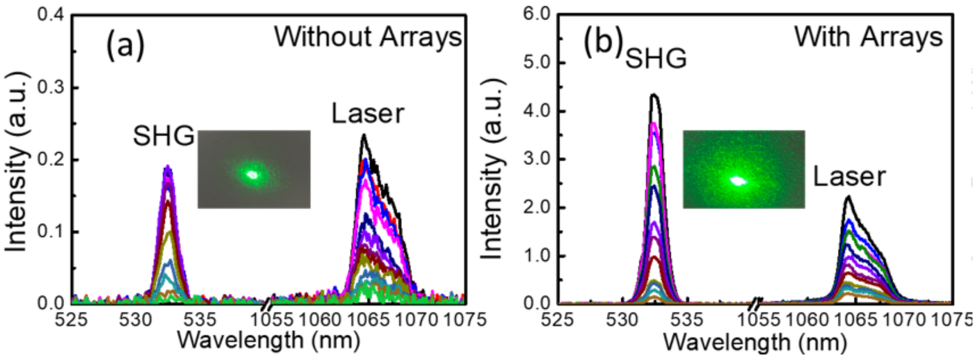

Figure S2 The spectra of fundamental frequency laser and SHG with the increase of excitation energy in (a) unpatterned sample and (b) surficial patterned sample
